# Metabolic and Structural Changes in Lower-Limb Skeletal Muscle Following Neuromuscular Electrical Stimulation: A Systematic Review

**DOI:** 10.1371/journal.pone.0069391

**Published:** 2013-09-03

**Authors:** Maurice J. H. Sillen, Frits M. E. Franssen, Harry R. Gosker, Emiel F. M. Wouters, Martijn A. Spruit

**Affiliations:** 1 Program Development Center, CIRO+, Center of Expertise for Chronic Organ Failure, Horn, The Netherlands; 2 NUTRIM School for Nutrition, Toxicology & Metabolism, Department of Respiratory Medicine, Maastricht University Medical Center+, Maastricht, The Netherlands; 3 CIRO+, Center of Expertise for Chronic Organ Failure, Horn, The Netherlands; West Virginia University School of Medicine, United States of America

## Abstract

**Background:**

Transcutaneous neuromuscular electrical stimulation (NMES) can be applied as a complementary intervention to regular exercise training programs. A distinction can be made between high-frequency (HF) NMES and low-frequency (LF) NMES. In order to increase understanding of the mechanisms of functional improvements following NMES, the purpose of this study was to systematically review changes in enzyme activity, muscle fiber type composition and muscle fiber size in human lower-limb skeletal muscles following only NMES.

**Methods:**

Trials were collected up to march 2012 and were identified by searching the Medline/PubMed, EMBASE, Cochrane Central Register of Controlled Trials, CINAHL and The Physical Therapy Evidence Database (PEDro) databases and reference lists. 18 trials were reviewed in detail: 8 trials studied changes in enzyme activities, 7 trials studied changes in muscle fiber type composition and 14 trials studied changes in muscle fiber size following NMES.

**Results:**

The methodological quality generally was poor, and the heterogeneity in study design, study population, NMES features and outcome parameters prohibited the use of meta-analysis. Most of the LF-NMES studies reported significant increases in oxidative enzyme activity, while the results concerning changes in muscle fiber composition and muscle size were conflicting. HF-NMES significantly increased muscle size in 50% of the studies.

**Conclusion:**

NMES seems to be a training modality resulting in changes in oxidative enzyme activity, skeletal muscle fiber type and skeletal muscle fiber size. However, considering the small sample sizes, the variance in study populations, the non-randomized controlled study designs, the variance in primary outcomes, and the large heterogeneity in NMES protocols, it is difficult to draw definitive conclusions about the effects of stimulation frequencies on muscular changes.

## Introduction

Regular exercise training programs consist of a combination of aerobic and strengthening exercises for developing and maintaining muscular endurance and strength, respectively [1]. Indeed, combined training modalities result in improvements in body composition and cardiorespiratory fitness. These improvements can partially be explained by intramuscular changes, such as an increased enzyme activity and an increased muscle fiber size [2], [3].

These intramuscular changes are dependent on the type of exercise training. Generally, aerobic exercise training results in increased levels of oxidative enzymes [4] and only a marginal increase in percentage type I fibers [5]; whereas resistance training results in increased levels of glycolytic enzymes [6] and an increase in percentage and size of type II fibers [7]–[10].

Neuromuscular electrical stimulation (NMES) can be applied as an complement intervention to voluntary exercise training [11]. NMES involves the application of an electric current through electrodes placed on the skin over the targeted muscles, thereby depolarizing motor endplates via the motor nerve and, in turn, inducing skeletal muscle contractions [12], [13]. NMES is composed of stimulation-rest cycles situated in regard to muscle motor points [14]. In contrast to voluntary muscle actions, NMES activates the muscle to a greater extent under identical technical conditions [15]. At identical levels of workload (10% of the quadriceps maximum isometric voluntary torque), the muscle reaches higher values in blood flow and oxygen consumption during NMES compared with voluntary contractions [15]. Moreover, a single session of NMES is sufficient to stimulate molecular-level responses, which are indicative of the initiation of myogenic processes in skeletal muscle, while an additional NMES session (a total of 14 minutes spread over 2 days), was sufficient to induce an increase in the concentration of total ribonucleic acid (RNA) [16], most likely representing an increase in muscle protein synthesis. There is sufficient evidence that NMES induced contractions differ physiologically compared to voluntary contractions [17]. In human studies contradictory findings on motor unit recruitment order have been found [18]. Some studies suggest preferential or selective activation of fast motor units with NMES [19], [20], whereas others suggest that motor unit recruitment during NMES reflects a non-selective, spatially fixed, and temporally synchronous pattern rather than in a reversal of the physiological voluntary recruitment order [17]. These diverse results could have been related to differences in protocols and stimulated muscles [14].

In daily clinical practice, lower-limb NMES improves skeletal muscle mass and function, exercise capacity and health status [21]–[23], particularly in subjects who are unable to perform or complete volitional exercise training programs. Therefore, NMES may be valuable in dyspneic and deconditioned patients with chronic organ failure due to the low metabolic load on the impaired cardio-respiratory system [23], [24].

NMES training sessions generally last 10–30 minutes during a 4- to 5-week period that involves 20–25 sessions to increase peripheral muscle function [14]. 2 types of NMES frequencies can be distinguished: high-frequency NMES (HF-NMES, >50 Hertz); and low-frequency NMES (LF-NMES, <20 Hertz) [13], [14], [25]–[27]. Frequencies of 50 Hertz and above induce a fused tetanus [28], [29] and generate higher torques than low frequencies [30]. The mechanisms by which NMES results in increased muscle strength or endurance are poorly understood. In isolated muscles in rats HF-NMES induces anabolic processes similar to resistance training (e.g. increased PKB-TSC2-mTor and protein synthesis) and LF-NMES similar to endurance training (AMPK-PG C1α activation) [31]. In humans, it is unknown which stimulation frequency is involved in the specific physiological and biochemical processes [13].

To date, narrative reviews have been published about the effects of NMES on gains in muscle performance, activation of motor units and/or muscle energetics [14], [26], [32], [33]. However, there is a broad diversity in NMES programmes, populations and outcomes which makes it difficult to interpret the conclusions. The effects of NMES on intramuscular changes have not been systematically reviewed yet. The purpose of this study is to systematically review changes in enzyme activity, muscle fiber type composition and muscle fiber size in human lower-limbs following a NMES programme. A distinction will be made between HF-NMES and LF-NMES, as well as in healthy volunteers, patients with chronic organ failure or orthopedic problems. Our hypothesis is that LF-NMES (<20 Hz) will primarily induce endurance training-like adaptations such as increased oxidative enzyme capacity and fiber type I proportion, whereas HF-NMES (>50 Hz) will primarily induce adaptations comparable to resistance training such as an increased glycolytic capacity, fiber type II proportion and muscle fiber size. Safety and the methodological quality of the trials will also be assessed.

## Methods

### Data sources and searches

We followed the procedures described in the PRISMA statement for reporting systematic reviews (online supplement) [34]. A broad computerized literature search was performed to identify relevant trials reported in the English language. We used the following databases: Medline/PubMed (from 1966), EMBASE (from 1974), Cochrane Central Register of Controlled Trials (from 1898), CINAHL (from 1982), and The Physical Therapy Evidence Database (PEDro) (from 1982). Trials were collected up to March 2012. Search terms were combinations of keywords related to neuromuscular electrical stimulation, lower-limb muscles, muscle mass and muscle metabolic profile. The exact search algorithm for Medline/PubMed can be found in Appendix 1. Similar search algorithms were used for the other databases. In addition, reference lists and citations of original articles were also scanned to identify additional articles that may contain information on the topic of interest.

### Data extraction

A pre-designed data abstraction form was used to obtain data on trial design and relevant results. For each article, characteristics of the study subjects were noted: a) the condition of the study population (healthy or primary diagnosis), gender and age; b) study design and NMES features (i.e. pulse duration, pulse frequency, duty cycle and pulse amplitude of the used current, training intensity, session time and duration in weeks); c) outcome measures, such as muscle enzyme activity (i.e. changes in oxidative and glycolytic enzymes), changes in muscle fiber type, changes in muscle fiber size and d) safety.

### Article selection

Articles were used for further analyses when they met the following eligibility criteria:

#### Types of studies

Randomized controlled trials (RCTs), controlled clinical trials (CCTs) and clinical trials were included. *A priori*, congress abstracts, reviews, editorials and case reports were considered ineligible.

#### Study subjects

Included were trials in which human lower-limb muscles were electrically stimulated. Reasons for exclusion were studies with subjects suffering from neurological disorders (e.g., hemiplegia or lesion of the spine) or smooth muscle problems (e.g., period of bladder dysfunction)

#### Types of interventions

Included were trials in which the muscles were stimulated transcutaneously at the muscle motor points with a stimulation frequency of <20 Hertz (LF-NMES) or >50 Hertz (HF-NMES), a minimum total session time of 120 minutes, a minimum of 3 sessions per week in a minimum of 4 weeks [13], [14], [18]. Trials were not excluded based on pulse duration, pulse amplitude or training intensity.

#### Types of outcome measures

In the reviewed publications the outcome measures were muscular activities of enzymes involved in oxidative or glycolytic energy metabolism, changes in fiber type composition and/or muscle fiber size following NMES.

### Assessment of methodological quality

The methodological quality of the identified trials was scored using the PEDro scale and is based on the Delphi list and “expert consensus” [35]. The PEDro scale consists of 11 criteria which receives either a “yes” or a “no”. Criterion 1 (‘Eligibility criteria’) is not used in the calculation of the PEDro score. All “yes” scores were summed resulting in a maximum score of 10 points [35]. A κ coefficient was used to measure the level of interrater reliability, using a method for comparing the level of reliability with categorical data along with their respective 95% confidence intervals [36]. Consensus was sought in case of disagreement. Trials with a PEDro score of ≥6 points were classified as “high-quality trials”, while trials with a PEDro score ≤5 points were classified as “low-quality trials” [37].

### Data analysis

The use of meta-analytic techniques for data-pooling was not possible, because of the heterogeneity in study types, study populations, wide diversity in NMES protocols (e.g., frequency, pulse duration, session time, total number of sessions) and/or outcome parameters (e.g., activity of different enzymes). Also, the technique for measuring muscle fiber size differed among the included studies. Therefore, the present authors were only able to systematically review the available peer-reviewed literature and to critically appraise the methodological quality and the overall findings.

## Results

### Search and selection

**Figure 1:**
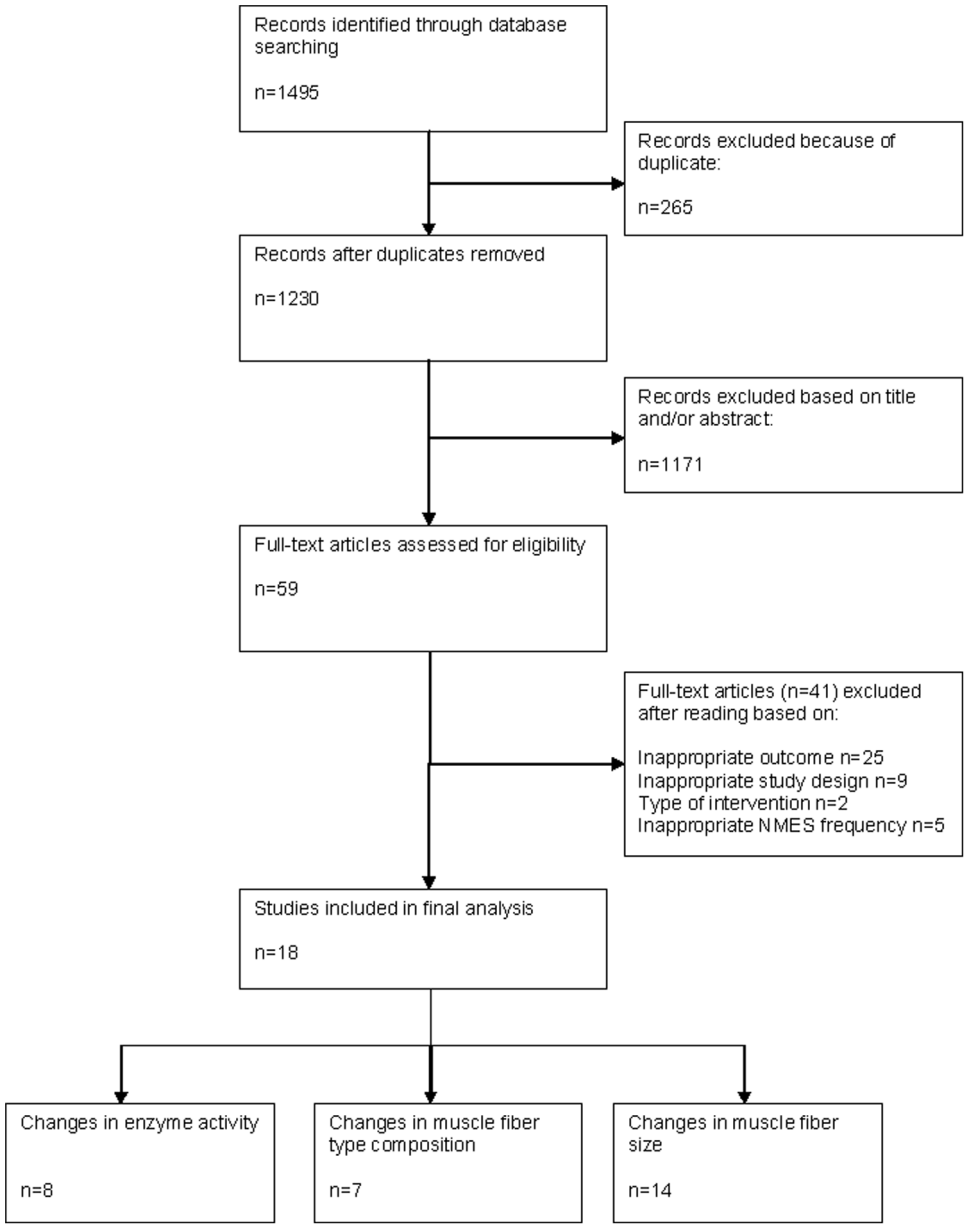
Screening and selection process of trials.

After removing duplicates, 1230 potentially relevant studies were identified by screening electronic databases. No trials were additionally identified by scanning reference lists. Of these trials, 1171 were excluded based on title and/or abstract. Of the remaining 59 trials, 41 trials were excluded after reading the full text based on type of intervention, outcome parameters and/or publication type. Finally, 18 [38]–[55] trials were included. 8 trials [39], [40], [46]–[48], [52]–[54] studied changes in enzyme activity, 7 trials [38], [40], [46]–[48], [52], [54] studied changes in muscle fiber type composition, and 14 trials [38], [40]–[45], [48]–[52], [54], [55] studied changes in muscle fiber size following NMES (figure 1).

A non-controlled experimental design was used in 6 studies, a controlled clinical trial was used in 3 studies and a randomized controlled design was used in 9 studies.

### Methodological quality of the trials (table 1)

The interrater reliability for the reviewers (MJHS and FMEF) was 0.74 (95% confidence interval, 0.68 to 0.80; p<0.001).

PEDro scores ranged from 2 to 9 points in the trials concerning changes in enzyme activity (median: 5.5 points). 4 trials (50%) [46]–[48], [54] scored >6 points on the PEDro scale. Of the trials studying muscle fiber type composition, PEDro scores ranged from 2 to 9 points (median: 6 points). 5 trials (71%) [38], [46]–[48], [54] scored >6 points on the PEDro scale. In the trials concerning changes in muscle fiber size, PEDro scores ranged from 2 to 9 points (median: 4 points). 5 trials (36%) [38], [48], [49], [54], [55] scored >6 points on the PEDro scale.

Overall, eligibility criteria [41], [42], [45], [48], [53], failure to conceal allocation, and blinding of the participants, therapists and outcome assessors were the most prevalent methodological shortcomings (table 1).

**Table 1:** Methodological quality (PEDro scale).

Study	1 Eligibility Criteria	2 Random allocation	3 Concealed allocation	4 Similarity at baseline	5 Blinding patients	6 Blinding therapists	7 Blinding assessors	8 Outcome >85% patients	9 Treatment as allocated or intention to treat	10 Between-group comparisons	11 Point measures and measures of variability	Quality (PEDro score)
**Healthy volunteers**												
Gauthier et al., 1992	Yes	No	No	No	No	No	No	Yes	Yes	No	Yes	3
Martin et al. 1994	No	No	No	Yes	No	No	No	No	No	No	Yes	2
Thériault et al., 1994	No	No	No	No	No	No	No	Yes	Yes	No	Yes	3
Thériault et al. 1996	Yes	No	No	Yes	No	No	No	Yes	Yes	Yes	Yes	5
Perez et al. 2002	No	Yes	No	Yes	No	No	No	Yes	Yes	Yes	Yes	6
Nuhr et al. 2003	Yes	Yes	Yes	Yes	Yes	No	Yes	No	No	Yes	Yes	7
Gondin et al., 2005	No	Yes	No	No	No	No	No	Yes	Yes	No	Yes	4
Gondin et al., 2011	Yes	No	No	No	No	No	No	No	Yes	No	Yes	2
Herrero et al., 2006	No	Yes	No	No	No	No	No	No	No	No	Yes	2
**Patients with COPD or CHF**												
Maillefert et al., 1998	Yes	No	No	Yes	No	No	No	No	No	No	Yes	2
Quittan et al., 2001	Yes	Yes	Yes	Yes	No	No	Yes	No	Yes	Yes	Yes	7
Jancik et al., 2002	Yes	No	No	No	No	No	No	Yes	Yes	No	Yes	3
Nuhr et al., 2004	Yes	Yes	Yes	Yes	Yes	No	No	Yes	Yes	Yes	Yes	8
Dal Corso et al., 2007	Yes	Yes	No	No	Yes	No	No	Yes	Yes	Yes	Yes	6
Vivodtzev et al., 2012	Yes	Yes	Yes	Yes	Yes	No	Yes	Yes	Yes	Yes	Yes	9
**Patients with orthopedic problems**												
Singer et al., 1986	Yes	No	No	No	No	No	No	Yes	Yes	No	No	2
Rebai et al., 2002	Yes	Yes	No	Yes	No	No	No	No	Yes	Yes	No	4
Walls et al., 2010	Yes	Yes	No	Yes	No	No	Yes	Yes	Yes	Yes	Yes	7

### NMES Protocols (table 2)

There was a substantial heterogeneity in the studied populations and NMES features (table 2). For example, 6 studies enrolled patients with chronic organ failure, divided in 2 studies [38], [54] including patients with chronic obstructive pulmonary disease (COPD) and 4 studies [43], [44], [47], [49] including patients with chronic heart failure (CHF). In 3 studies [50], [51], [55] the subjects had orthopedic problems of the lower limbs and in 9 studies [39]–[42], [45], [46], [48], [52], [53] healthy volunteers were enrolled.

**Table 2:** Study characteristics and NMES features.

Authors	Study design	Subjects	Outcome parameters	Stimulated Muscles	Frequency (Hz)	Pulse duration (ms)	On-off time (s)	Pulse amplitude	Session time	Total time
**Healthy volunteers**										
Gauthier et al., 1992	Experimental design	Healthy volunteers	Enzyme activity	QF	8	0.3	55: 2	N/A	3 hours/day	6 days/week, 6 weeks
		n = 26 (16 men)								
		mean age (years) ±SD): women: 26±4 men: 27±3								
Thériault et al. 1994	Experimental design	Healthy volunteers	Enzyme activity	QF	8	N/A	N/A	Very visible contraction	8 hours/day	6 days/week, 8 weeks
		n = 8 (7 men)								
		age (years): range 18-26								
Thériault et al. 1996	CCT	Healthy volunteers	Enzyme activity, muscle fiber CSA and muscle fiber composition	QF	8	0.3	55: 2	Very visible contraction	3 hours/day	6 days/week, 6 weeks
	NMES *versus* active subjects *versus* trained cyclists	n = 35 (number of men not reported)								
		age (years, range): 17–35								
Pérez et al., 2002	RCT	Healthy volunteers	Enzyme activity, muscle fiber CSA and muscle fiber composition	QF	45-60	0.3	12: 8	Maximum tolerance level	30 min	3 days/week, 6 weeks
	NMES *versus* no treatment	n = 15 (all men)								
		mean age (years, ±SD): 22±5								
Nuhr et al., 2003	RCT	Healthy volunteers	Enzyme activity and muscle fiber composition	QF and HM	15	0.5	2: 4	Maximum tolerance level	2 hours/day	2 sessions/ day, 7 days/week, 10 weeks
	NMES *versus* sham-stimulation (no evoked contractions)	n = 20 (all men)								
		mean age (years, ±SD): NMES group:30±1 Sham group: 34±3								
Gondin et al., 2011	Experimental design	Healthy volunteers	Enzyme activity, muscle fiber CSA and muscle fiber composition	QF	75	0.4	6.25: 20	61mA (sedentary group) and 81mA (active group)	20 min	3 days/week, 8 weeks
		n = 10 (all men)								
		mean age (years, ±SD): 26±3								
Martin et al., 1994	CCT	Healthy volunteers (physical education students)	Whole muscle CSA	TS	70	0.2	5: 15	63% of MVC	10 min	3 times/week, 4 weeks
	NMES *versus* no treatment	n = 12 (number of men not reported)								
		mean age (years, ±SD): NMES group: 24.2±1.8 control group: 23.2±2.6								
Gondin et al., 2005	RCT	Healthy volunteers (students)	Whole muscle CSA	QF	75	0.4	6: 20	Maximum tolerance level	18 min	4 days/week, 8 weeks
	NMES *versus* no treatment	n = 20 (all men)								
		mean age (years, ±SD): NMES group 23.5±5.0 Control group 24.3 ±1.6								
Herrero et al., 2006	RCT	Healthy volunteers (physical education students)	Whole muscle CSA	QF	120	0.4	3: 30	Maximum tolerance level	34 min	4 days/week, 4 weeks
	NMES *versus* plyometric training *versus* NMES and plyometric training *ersus* no treatment	n = 40 (all men)								
		mean age (years, ±SD): NMES group 19.4±0.4) Plyometric training group: 20.8±0.6 NMES/plyometric training group: 21.4±0.9 Control group 20.6±0.6								
**Patients with CHF or COPD**										
Nuhr et al., 2004	RCT	Patients with severe CHF	Enzyme activity and muscle fiber composition	QF and HM	15	0.5	2: 4	Till 25-30% of MVC force	2 hours	2 sessions/ day, 7 days/week, 10 weeks
	NMES *versus* sham-stimulation (no evoked contractions)	n = 34 (29 men)								
		mean age (years, ±SD): NMES group: 53±7 Sham group: 53±13								
Vivodtzev et al., 2012	RCT	Patients with severe COPD	Enzyme activity, muscle fiber CSA and muscle fiber composition	QF and TS	50	0.4	6: 16	Maximum tolerance level (mean pulse amplitude at beginning 20mA and at end 31mA)	1 hour (35 min QF and 25 min TS)	5 days/week, 6 weeks
	NMES versus sham-stimulation	n = 20 (13 men)								
		median age (years, (IQR): NMES group: 59 (57-59) sham group: 67 (59-72)								
Dal Corso et al., 2007	Prospective cross-over single-blinded RCT	Patients with moderate to severe COPD	Muscle fiber CSA and muscle fiber composition	QF	50	0.4	first week 2:10 week 6 10: 20	Ranging 10–25 mA, increased weekly with 5 mA	30 min	5 days/week, 6 weeks
	NMES *versus s*ham-stimulation	n = 17 (16 men)								
		mean age (years, ±SD): 65.9±6.8								
Maillefert et al., 1998	Experimental design	Patients with CHF	Whole muscle CSA	QF and TS	10	0.2	20: 20	Maximal tolerance level (maximal amplitude 60 mA)	1 hour	5 days/week, 5 weeks
		n = 19 (16 men)								
		mean age (years±SD): 57.8±8.7)								
Quittan et al., 2001	RCT	Patients with CHF	Whole muscle CSA	QF and HM	50	0.7	2: 6	Till 25–30% of MVC	30–60 min	5 days/week, 8 weeks
	NMES *versus* usual care	n = 21(15 men)								
		mean age (years, ±SD) Stimulation group: 59±6 Control group: 57±8								
Jancik et al., 2002	Experimental design	Patients with CHF	Whole muscle CSA	QF and TS	10	0.2	20: 20	N/A (maximum 60 mA)	1 hour	5 days/week, 5 weeks
		n = 12 (number of men not reported)								
		mean age (years, ±SD) 56±9								
**Patients with orthopedic problems**										
Singer et al. 1986	Experimental design	Patients with lower limb or knee joint injury/surgery	Whole muscle CSA	QF	50, 50, 100	0.35, 0.25, 0.075	8: 10	Comfortable stimulus producing tetanic contractions	15 min	7 days/week, 4 weeks
		n = 35 (all men)								
		mean age (years, ±SD): 34.4±5.8								
Rebai et al., 2002	RCT	Patients with isolated ACL injury	Whole muscle CSA	QF	80 and 20	0.3	80 Hz 15: 75 and 20 Hz 15 10	80 Hz 35% of MVC and 20 Hz 25% of MVC	80 Hertz 54 min and 20 Hertz 60 min	5 days/week, 12 weeks
	High-frequency NMES *versus* Low-frequency NMES	n = 10 (all men)								
		mean age (years,±SD) 20 Hz group: 27±4.76 80 Hz group 25±2.5								
Walls et al., 2010	RCT	Patients with end-stage osteoarthritis preoperative TKA	Whole muscle CSA	QF	50	0.4	5: 10	Maximum tolerance level (maximum intensity 70 mA)	20 min	5 days/week, 6 weeks
	NMES *versus* muscle strengthening exercises	n = 14 (4 men)								
		mean age (years, ±SD): NMES group 64.4±8.0 Control group 63.2±11.4								

ACL  =  anterior cruciate ligament; CHF  =  chronic heart failure; CCT  =  controlled clinical trial; COPD  =  chronic obstructive pulmonary disease; CSA  =  cross-sectional area; HM  =  hamstrings muscles; Hz  =  hertz; ICU  =  intensive care unit; IQR  =  interquartile range; mA  =  milliampere; min  =  minutes; ms  =  milliseconds; MVC  =  maximal voluntary contraction; NMES  =  neuromuscular electrical stimulation; N/A  =  not available; s =  seconds; QF  =  quadriceps femoris; RCT  =  randomized controlled trial; SD  =  standard deviation; TKA  =  total knee arthroplasty; TS  =  triceps surae.

Different lower-limb muscles were stimulated in the identified trials: quadriceps femoris muscles [38]–[42], [48], [50]–[53], [55], calf muscles [45], quadriceps femoris muscles combined with calf muscles [43], [44], [54], or quadriceps femoris muscles combined with hamstrings [46], [47], [49].

All trials used biphasic impulse current forms ranging from 8 to 20 and 50 to 120 Hertz. Pulse duration, not reported in 1 study [53], ranged between 200 and 700 µs. Duty cycle, not reported in 1 study [53], ranged between 3 seconds on, 30 seconds off to 55 seconds on, 2 seconds off. Pulse amplitude, not reported in 1 study [39], varied between 10 mA until the individual's maximum level of toleration. Session time varied between 10 minutes and 8 hours, 1 to 2 times a day. The total number of sessions varied between 12 and 140 (table 2) between 4 and 10 weeks. The total session time ranged from 2 to 384 hours.

### Safety

Safety was not reported in 13 trials. In 3 trials [38], [47], [49] no relevant side effects or adverse events were reported. Only once [49] a delayed onset muscle soreness was reported and one trial explicitly reported the absence of serious discomfort in the stimulated subjects [41]. Finally, in 1 trial [54] 1 study subject withdrew because of discomfort during NMES.

### Changes in enzyme activity following NMES

5 trials studied changes in enzyme activity following LF-NMES [39], [46], [47], [52], [53] and 3 trials following HF-NMES [40], [48], [54] (tables 3 and 4). The study subjects consisted of healthy volunteers [39], [40], [46], [48], [52], [53], patients with severe CHF [47] or severe COPD [54]. Enzyme activity was determined using muscle biopsies in the vastus lateralis of the quadriceps muscle in all studies.

**Table 3:** Enzyme activity following LF-NMES.

Study	Subjects	Frequency (Hz)	Enzyme activity	
			Oxidative enzymes	Glycolytic enzymes
Gauthier et al., 1992	Healthy volunteers	8	Citrate synthase (females 31%↑, males 18%↑) Mean changes in females of 3.4 µmol*min-1.g wet wt-1 muscle (11.1 µmol*min-1.g wet wt-1 muscle before NMES vs. 14.5 µmol*min-1.g wet wt-1 muscle after NMES). Mean changes in males of 2.3 µmol*min-1.g wet wt-1 muscle (12.8 µmol*min-1.g wet wt-1 muscle before NMES vs. 15.1 µmol*min-1.g wet wt-1 muscle after NMES).	Glyceraldehydephosphate dehydrogenase (females 0% change, males 7%↓) Mean changes in females of 4 µmol*min-1.g wet wt-1 muscle (446 µmol*min-1.g wet wt-1 muscle before NMES vs. 450 µmol*min-1.g wet wt-1 muscle after NMES). Mean changes of -42 µmol*min-1.g wet wt-1 muscle (615 µmolxmin-1.g wet wt-1 muscle before NMES vs. 573 µmolxmin-1.g wet wt-1 muscle after NMES).
			Cytochrome oxidase (females 19%↑, males 16%↑) Mean changes in females of 1.5 µmol*min-1.g wet wt-1 muscle, (8.0 µmol*min-1.g wet wt-1 muscle before NMES vs. 9.5 µmol*min-1.g wet wt-1 muscle after NMES). Mean changes in males of 1.4 µmol*min-1.g wet wt-1 muscle (8.7 µmol*min-1.g wet wt-1 muscle before NMES vs. 10.1 µmol*min-1.g wet wt-1 muscle after NMES).	Phosphofructokinase (females 8%↓, males 10%↓) Mean changes in females of -5 µmol*min-1.g wet wt-1 muscle (63 µmolxmin-1.g wet wt-1 muscle before NMES vs. 58 µmolxmin-1.g wet wt-1 muscle after NMES). Mean changes in males of -8 µmol*min-1.g wet wt-1 muscle (78 µmol*min-1.g wet wt-1 muscle before NMES vs. 70 µmol*min-1.g wet wt-1 muscle after NMES).
			Hydroxyacyl CoA dehydrogenase (HADH) (females 30%↑, males 7%↑) Mean changes in females of 4.8 µmol*min-1.g wet wt-1 muscle (15.80 µmol*min-1.g wet wt-1 muscle before NMES vs. 20.6 µmol*min-1.g wet wt-1 muscle after NMES). Mean changes in males of 1.3 µmol*min-1.g wet wt-1 muscle (18.7 µmol*min-1.g wet wt-1 muscle before NMES vs. 21.0 µmol*min-1.g wet wt-1 muscle after NMES).	Hexokinase (females 36%↑, males 9%↑) Mean changes in females of 0.4 µmol*min-1.g wet wt-1 muscle (1.1 µmol*min-1.g wet wt-1 muscle before NMES vs. 1.5 µmolxmin-1.g wet wt-1 muscle after NMES). Mean changes in males of 0.1 µmol*min-1.g wet wt-1 muscle (1.1 µmolxmin-1.g wet wt-1 muscle 1 before NMES vs. 1.2 µmol*min-1.g wet wt-1 muscle after NMES).
Theriault et al., 1994	Healthy volunteers	8	Citrate synthase (13%↑) Changes of 10.6 µmol*min-1.g wet wt-1 before NMES vs. 13. µmol*min-1.g wet wt-1 after 4 weeks NMES vs. 12.0 µmol*min-1.g wet wt-1 after 8 weeks NMES.	Phosphofructokinase (23%↓) Changes of 51.9 µmol*min-1.g wet wt-1 before NMES vs. 41.0 µmol*min-1.g wet wt-1 after 4 weeks NMES vs. 40.6 µmol*min-1.g wet wt-1 after 8 weeks NMES.
			Cytochrome oxidase (30%↑) Changes 7 µmol*min-1.g wet wt-1 before NMES vs. 9.00 µmol*min-1.g wet wt-1 after 4 weeks NMES vs. 9.1 µmol*min-1.g wet wt-1 after 8 weeks NMES.	Glyceraldehydephosphate dehydrogenase (11%↓) Changes of 457 µmol*min-1.g wet wt-1 before NMES vs. 400 µmol*min-1.g wet wt-1 after 4 weeks NMES vs. 407 µmol*min-1.g wet wt-1 after 8 weeks NMES.
			HADH (12%↑) Significant changes of 15.3 µmol*min-1.g wet wt-1 before NMES vs. 18.4 µmol*min-1.g wet wt-1 after 4 weeks NMES vs. 17.1 µmol*min-1.g wet wt-1 1 after 8 weeks NMES.	
Theriault et al., 1996	Healthy volunteers	8	Citrate synthase (18%↑) Mean changes of 2.1 µmol*min-1.g wet wt-1 (11.6 µmol*min-1.g wet wt-1 before NMES vs. 13.7 µmolxmin-1.g wet wt-1 after NMES). Citrate synthase activity is significant higher in trained cyclists compared with NMES group and active subjects. Citrate synthase activity is significant higher in active subjects compared with NMES group.	
Nuhr et al., 2003	Healthy volunteers	15	Citrate synthase (9%↑) Mean changes in citrate synthase in the NMES-group compared with sham-stimulation (p<0.05). NMES-group: mean changes in citrate synthase of 1.1 µmol*min-1.g wet wt-1 (12.7 µmol*min-1.g wet wt-1 before NMES vs. 13.8 µmol*min-1.g wet wt-1 after NMES). Sham-stimulation group: changes are not reported.	Glyceraldehydephosphate dehydrogenase (7%↓) Mean changes in glyceraldehydrephosphate dehydrogenase in the NMES-group compared with sham-stimulation (p<0.05). NMES-group: mean changes in glyceraldehydephosphate dehydrogenase of -21.3 µmol*min-1.g wet wt-1 (324.7 µmol*min-1.g wet wt-1 before NMES vs. 303.4 µmol*min-1.g wet wt-1 after NMES). Sham-stimulation group: changes are not reported. Glyceraldehydephosphate dehydrogenase (15%↓) Mean changes in glyceraldehydrephosphate dehydrogenase in the NMES-group compared with sham-stimulation (p<0.05). NMES-group: mean changes in glyceraldehydephosphate dehydrogenase of -41 units per gram wet wt-1 (277 units per gram wet wt-1 before NMES vs. 236 units per gram wet wt-1 after NMES). Sham-stimulation group: mean changes in glyceraldehydephosphate dehydrogenase of 12 units per gram wet wt-1 (277 units per gram wet wt-1 before NMES vs. 289 units per gram wet wt-1 after NMES).
Nuhr et al., 2004	Patients with severe CHF	15	Citrate synthase (30%↑) Mean changes in citrate synthase in the NMES-group compared with sham-stimulation (p<0.05). NMES-group: mean changes in citrate synthase of 1.0 units per gram wet wt-1 (3.3 units per gram wet wt-1 before NMES vs. 4.3 units per gram wet wt-1 after NMES). Sham-stimulation group: mean changes in citrate synthase of -0.3 units per gram wet wt-1 (3.4 units per gram wet wt-1 before NMES vs. 3.1 units per gram wet wt-1 after NMES).	Glyceraldehydephosphate dehydrogenase (15%↓) changes in glyceraldehydrephosphate dehydrogenase in the NMES-group compared with sham-stimulation (p<0.05). NMES-group: mean changes in glyceraldehydephosphate dehydrogenase of -41 units per gram wet wt-1 (277 units per gram wet wt-1 before NMES vs. 236 units per gram wet wt-1 after NMES). Sham-stimulation group: mean changes in glyceraldehydephosphate dehydrogenase of 12 units per gram wet wt-1 (277 units per gram wet wt-1 before NMES vs. 289 units per gram wet wt-1 after NMES).

**Table 4:** Enzyme activity following HF-NMES.

Study	Subjects	Frequency (Hz)	Enzyme activity	
			Oxidative enzymes	Glycolytic enzymes
Perez et al, 2002	Healthy volunteers	45–60	Succinate dehydrogenase (16%↑) Succinate dehydrogenase activity increased ?16% vs. control group.	
Gondin et al., 2011	Healthy volunteers	75	Active group post-NMES vs Active group pre-NMES: NADH-ubiquinone oxireductase ↑ Ubiquinol cyt C reductase ↑ Enoyl CoA hydratase	Active group post-NMES vs Active group pre-NMES: β-enolase ↑
			Sedentary group post-NMES vs sedentary group pre-NMES: Acyl CoA dehydrogenase ↓ Pyruvate dehydrogenase ↑ Isocitrate dehydrogenase ↑ Ubiquinol cyt C reductase ↑	Sedentary group post-NMES vs sedentary group pre-NMES: Phosphofructokinase ↓ β-enolase =
Vivodtzev et al., 2012	Patients with severe COPD	50	No significant changes in enzyme activity after training Citrate synthase (2%↑) NMES-group: mean changes in citrate synthase of 13.5 (+5.1) µmol*min-1.g wet wt-1 before NMES vs.13.2 (+8.2) µmol*min-1.g wet wt-1 after NMES. Sham-stimulation group: mean changes in citrate synthase (17%↓) of 9.0 (+2.2) µmol*min-1.g wet wt-1 before NMES vs.10.8 (+2.4) µmol*min-1.g wet wt-1 after NMES.	
			HADH (7%↓) NMES-group: mean changes in HADH of 4.2 (+1.2) µmol*min-1.g wet wt-1 before NMES vs.3.9 (+1.1) µmol*min-1.g wet wt-1 after NMES. Sham-stimulation group: mean changes in HADH of 3.6 (+1.2) µmol*min-1.g wet wt-1 before NMES vs.3.6 (+0.8) µmol*min-1.g wet wt-1 after NMES.	

### Changes in oxidative enzymes in healthy volunteers

Levels of oxidative enzymes generally increased following LF-NMES (table 3) and following HF-NMES (table 4).

#### Citrate synthase

Citrate synthase (CS), a marker enzyme for the tricarboxylic acid cycle (Krebs cycle), was an outcome parameter in 4 LF-NMES trials [39], [46], [52], [53]. In 3 trials [39], [52], [53] CS increased compared to baseline (9 to 31%) and in 1 trial [46] CS increased compared to sham-stimulation.

#### Isocitrate dehydrogenase

Isocitrate dehydrogenase, another enzyme that participates in the tricarboxylic acid cycle, increased significantly following HF-NMES compared to baseline [40].

#### 3-Hydroxylacyl-CoA dehydrogenase (HADH)

HADH, a key enzyme of ß-oxidation of fatty acids, increased significantly following LF-NMES compared to baseline in 2 trials in healthy volunteers (7–30%) [39], [53]. Contradictionary, in a HF-NMES trial [40] HADH decreased.

#### Enoyl CoA hydratase

Enoyl CoA hydratase, an enzyme that participates in the ß-oxidation of fatty acids, increased significantly following HF-NMES compared to baseline [40].

#### NADH-ubiquinone oxidoreductase

NADH-ubiquinone oxidoreductase, complex I of the electron transport chain, increased significantly following HF-NMES compared to baseline [40].

#### Succinate dehydrogenase

Succinate dehydrogenase, an enzyme that participates in both the tricarboxylic acid cycle and in complex II of the electron transport chain, increased significantly following HF-NMES compared to baseline and increased 16% compared to controls [48].

#### Ubiquinol-cytochrome c reductase

Ubiquinol cyt C reductase, complex III of the electron transport chain, increased significantly following HF-NMES compared to baseline [40].

#### Cytochrome c oxidase

Cytochrome c oxidase, complex IV of the electron-transfer chain metabolism, increased significantly following LF-NMES compared to baseline (16 to 19%) [39], [53].

#### Pyruvate dehydrogenase

Pyruvate dehydrogenase, an enzyme which contributes to linking the glycolysis metabolic pathway to the citric acid cycle and releasing energy via NADH, increased significantly following HF-NMES compared to baseline [40].

### Changes in oxidative enzymes in patients with CHF or COPD

Levels of CS increased following LF-NMES (15 Hertz) compared to sham-stimulation in patients with severe CHF [47] and did not change following HF-NMES (50 Hertz) in patients with severe COPD [54].Levels of HADH did not change following HF-NMES (50 Hertz) in patients with severe COPD [54].

### Changes in glycolytic enzymes in healthy volunteers

Levels of glycolytic enzymes generally did not change or decreased following LF-NMES or HF-NMES respectively (tables 3 and 4).

#### Phosphofructokinase (PFK)

Levels of PFK, a glycolytic enzyme that catalyses the phosphorylation of fructose phosphate, decreased or did not change compared to baseline following LF-NMES or HF-NMES (variation from baseline was between −11 and 0%) [39], [40], [53].

#### Glyceraldehyde 3-phosphate dehydrogenase (GAPDH)

Concentrations of GAPDH, a marker enzyme of anaerobic energy metabolism by catalysing the sixth step of glycolysis, decreased significantly in 1 LF-NMES trial [46] compared with sham-stimulation (variation from baseline was -15%). Levels of GAPDH did not change in 2 LF-NMES trials [39], [53] compared to baseline.

#### Hexokinase

Hexokinase, a key glycolytic enzyme, increased significantly in females (36%) and did not change in males following LF-NMES compared to baseline [39].

#### Β-enolase

Β-enolase, which catalyses the glycolysis of 2-phosphoglycerate to phosphoenolpyruvate, did not change in a sedentary group of healthy young men following HF-NMES compared to baseline, but increased in an active group of healthy young men following HF-NMES compared to baseline [40].

### Changes in glycolytic enzymes in patients with CHF

In patients with severe CHF levels of GAPDH decreased significantly in 1 LF-NMES trial [47] compared with sham-stimulation (variation from baseline was −15%).

### Skeletal muscle fiber type composition following NMES

3 trials [46], [47], [52] studied skeletal muscle fiber type composition following LF-NMES and 4 trials [38], [40], [48], [54] following HF-NMES (table 5). The study subjects consisted of healthy volunteers [40], [46], [48], [52], patients with severe CHF [47] and patients with severe COPD [38], [54].

**Table 5:** Skeletal muscle fiber type composition following NMES.

Study	Subjects	Frequency	Changes in muscle fiber type composition			
			Type I fibers	Type II fibers	Type IIa fibers	Type IIb/x fibers
Theriault et al., 1996	Healthy volunteers	8	=		19%↑	32%↓
Nuhr et al., 2003	Healthy volunteers	15	15%↑		=	22%↓
Perez et al, 2002	Healthy volunteers	45–60	15%↓		63%↑	88%↓
Gondin et al., 2011	Healthy volunteers	75	Active group 20%↑ Sedentary group 96%↑		Active group 9%↓ Sedentary group 42%↑	Sedentary group 79%↓
Nuhr et al., 2004	Patients with CHF	15	19%↑		=	19%↓
Dal Corso et al., 2007	Patients with moderate to severe COPD	50	4%↓	=		
Vivodtzev et al., 2012	Patients with severe COPD	50	21%↓		=	=

Data are shown as variation from baseline.

### Healthy volunteers

#### Type I fibers

Proportion of type I fibers increased in 1 LF-NMES trial (15%) [46] and 1 HF-NMES trial (active group 20% and sedentary group 96%) [40], and did not change in 1 LF-NMES trial [52]. This fiber type decreased in 1 trial following HF-NMES (−15%) [48].

#### Type II fibers

Type IIa fibers proportions increased following LF-NMES (19%) [52] and HF-NMES (63%) [48]. In another HF-NMES trial this fiber type increased in the sedentary group (42%) and decreased in the active group (9%) [40].

Type IIx fibers proportions decreased in 2 LF-NMES trials (22% and 32%) [46], [52] and 2 HF-NMES trials (79% and 88%) [40], [48].

### Patients with CHF or COPD

Proportion of type I fibers increased (19%) following LF-NMES [47] and decreased (4% and 21%) following HF-NMES [38], [54], type II proportions did not change following HF-NMES [38].

Type IIa proportions did not change in patients with CHF following LF-NMES [47] and these fiber type proportions remained unchanged compared to controls in patients with COPD following HF-NMES [54]. Type IIx fibers decreased following LF-NMES (19%) [47] and did not change following HF-NMES compared to a control group [54].

### Changes in muscle size following NMES

Different techniques were used to determine changes in whole muscle cross-sectional area (CSA) or muscle fiber CSA following NMES (tables 6, 7 and 8). Muscle fiber CSA was measured by percutaneous needle biopsy of the vastus lateralis muscle [38], [40], [48], [52], [54]. Whole muscle CSA was measured by computed tomography [45], [49], [51], magnetic resonance imaging (MRI) [43], [44], [50], [55], ultrasonography [41] or circumference and skinfold measurements [42]. Maillefert and colleagues determined the total volume of the soleus muscles and gastrocnemius muscles by calculated muscle volume from serial CSAs measured by MRI [44].3 trials used LF-NMES [43], [44], [52] and 11 trials used HF-NMES [38], [40]–[42], [45], [48]–[51], [54], [55].

**Table 6:** Changes in muscle fiber size following NMES in healthy people.

Study	Frequency (Hz)	Type I CSA	Type II CSA	Whole muscle CSA/muscle fiber CSA
Theriault et al., 1996	8	Mean changes of CSA of type I fibers before 5437±1170 μm^2^ versus 5791±1381 μm^2^ after NMES.	Mean changes of CSA of type IIa fibers before 5568±1318 μm^2^ versus 6041±1515 μm^2^ after NMES.	No significant changes in CSA of the muscles before and after NMES.
			Mean changes of CSA of type IIx fibers before 4539±1314 μm^2^ versus 4850±1730 μm^2^ after NMES.	
Perez et al, 2002	45–60			CSA of the muscles increased (?14%) compared with controls (p<0.05).
Gondin et al, 2011	75			CSA of both fiber types increased after NMES, which was higher in type II fibers (+23%) compared with type I fibers (+12%).
Martin et al., 1994	70			Total CSA of the muscles was similar before and after NMES Mean CSA values in the triceps surae were 50.80+5.2 cm^2^ before NMES and 50.80+4.8 cm^2^ after NMES.
Gondin et al, 2005	75			CSA increased significantly in the NMES group compared with control group. CSA increased significantly (6.0+2%, p<0.001) in the NMES group compared with baseline.
Herrero et al, 2006	120			CSA increased significantly (9.0%, p<0.01) in the NMES group compared with baseline.

**Table 7:** Changes in muscle fiber size following NMES in patients with CHF or COPD.

Study	Frequency (Hz)	Type I CSA	Type II CSA	Whole muscle CSA/muscle fiber CSA
Mailllefert et al., 1998	10			Total volume of soleus muscles and gastrocnemius muscles increased significantly. Mean changes of total volume of soleus muscles before 319±42.9 cm^3^ versus 338±52.5 cm^3^ and gastrocnemius muscles before 259.4±58 cm^3^ versus 273.4±74 cm^3^ after NMES.
Jancik et al, 2002	10			Muscle mass volumes of gastrocnemius muscles increased significantly and of soleus muscles no significant differences were reported. Mean changes of total volume of gastrocnemius muscles before 254.3±47 cm^3^ versus 278.6±38 cm^3^ after NMES.and of soleus muscles before 315.2±65 cm^3^ versus 331.5±44 cm3 after NMES.
Quittan et al, 2001	50			CSA increased significantly (p<0.001) in the NMES group compared with the control group (p = 0.009). NMES group: Mean changes of CSA before 98.5±27.6 cm^2^ versus 111.3±24.2 cm^2^ after 8 weeks. Control group: Mean changes of CSA before 104.4±21.6 cm^2^ versus 106.4±22.8 cm^2^ after 8 weeks.
Dal Corso et al., 2007	50	Mean changes of CSA of type I fibers before 4610±1808 μm^2^ versus 4009±1329 μm^2^ after NMES.	Mean changes of CSA of type II fibers before 3786±1294 μm^2^ versus 4119±936 μm^2^ after NMES.	CSA of the muscles was similar before and after NMES.
Vivodtzev et al, 2012	50	Mean changes of CSA of type I fibers before 4636±722 μm^2^ versus 5129±969 μm^2^ after NMES.	Mean changes of CSA of type IIa fibers before 3423±397 μm^2^ versus 3673±545 μm^2^ after NMES.	No statistically significant changes in CSA between groups. Mean changes of CSA of all type fibers before 3488±450 μm^2^ versus 4061±735 μm^2^ after NMES
		Mean changes of CSA of type I fibers before 5252±505 μm^2^ versus 4818±422 μm^2^ after sham-stimulation	Mean changes of CSA of type IIa fibers before 4653±367 μm^2^ versus 3913±502 μm^2^ after sham-stimulation.	Mean changes of CSA of all type fibers before 4720±429 μm^2^ versus 4046±4530 μm^2^ after sham-stimulation.
			Mean changes of CSA of type IIx fibers before 2406±312 μm^2^ versus 3380±854 μm^2^ after NMES.	
			Mean changes of CSA of type IIx fibers before 4206±607 μm^2^ versus 4046±453 μm^2^ after sham-stimulation.	

**Table 8:** Changes in muscle fiber size following NMES in patients with orthopedic problems.

Study	Frequency (Hz)	Type I CSA	Type II CSA	Whole muscle CSA/muscle fiber CSA
Walls et al, 2010	50			CSA increased 7.4% following NMES (p = 0.036).
Singer et al., 1986	50–100			No significant changes in CSA.
Rebai et al, 2002	80 and 20			No significant differences in deficit in muscle volume between the groups were reported. At 12 weeks, the rate of recuperation was in the 20 Hzgroup 93% and in the 80 Hz group 89%.

#### Healthy people

Muscle fiber CSA did not change following 1 LF-NMES trial [52] and 1 HF-NMES trial [48]. Following another HF-NMES trial muscle fiber CSA increased, in both type I and type II fibers [40] (table 6). Whole muscle CSA was studied in 3 HF-NMES trials [41], [42], [45] and did not change in 1 trial [45] and increased in 2 trials [41], [42].

#### Patients with CHF or COPD

Following HF-NMES muscle fiber CSA did not change in one trial [38] and increased compared to sham stimulation in another trial [54] (table 7). Whole muscle CSA increased following LF-NMES [43], [44] and HF-NMES [49] (table 7).

#### Patients with orthopedic problems

Whole muscle CSA increased significantly in 1 HF-NMES trial [55] and did not change in two other HF-NMES trials [50], [51] (table 8).

## Discussion

This is the first systematic review on the effects of lower-limb NMES on intramuscular changes in the human lower-limb muscles. Most of the studies reported a significant increase in oxidative enzymes following LF-NMES. There are obvious changes in skeletal muscle fiber type composition following NMES. Indeed, LF-NMES seems to increase percentage of type I and IIa fibers, whereas fiber type composition following HF-NMES shows conflicting results. Both NMES protocols showed conflicting results in changes in muscle fiber size and total muscle volume. Heterogeneity in study design, study population, NMES features and outcome parameters prohibits the use of meta-analysis.

### Methodological considerations

Overall, the methodological quality of the included trials was poor (median score 4 points). None of the 18 included trials had a perfect score on the PEDro scale (table 2). In fact, only 7 trials (39%) were of high-quality. Eligibility criteria were not specified in 4 trials (22%) and a control group was lacking in 6 trials (33%). Other methodological considerations were the limited number of study subjects (n = 8 to n = 40), the low mean age (38 years) and the fact that most subjects studied were men. Elderly subjects may respond differently on anabolic training stimulus compared to younger subjects [56]. Moreover, gender-differences exist in fiber type distribution and mean CSA [57]. So, the internal and external validity of the findings of the reviewed trials were limited.

### NMES protocols

NMES protocols varied tremendously among the included trials. Pulse duration, if reported, ranged between 200 and 700 µs. Pulse duration of 300–400 µs is recommended for large muscle groups, such as the quadriceps muscles and calf muscles [58]. It remains currently unknown which duty cycle is optimal for effective treatment.

While throughout the literature a wide variety of protocols are used, there seems to be at least some agreement on the use of biphasic symmetrical pulses that last between 100 and 500 µs and are delivered at a pulse rate of 10–100 Hertz. Pulse rates between 10–50 Hz are used in patients with CHF and in patients with COPD with positive improvements in exercise capacity and health status [23], whereas pulse rates between 50–100 Hz are mostly recommended for gains in muscle performance [14], [18]. Such pulses are widely accepted as being well tolerated.

The stimulus intensity varied among the included trials, from a comfortable stimulus till maximum tolerance level. These diverse results could have been related to differences in protocols and stimulated muscles. It is strongly recommended that pulses are delivered at the highest tolerable pulse amplitude [22]. Another common procedure is to quantify isometric maximal voluntary contraction (MVC) force at the beginning of a NMES session, and subsequently express the level of each electrically elicited contraction as a percentage of the MVC force [18].

The duration of the NMES programmes varied between 10 minutes to 8 hours/day, 1 to 2 sessions/day, 3 to 7 days/week for 4 to10 weeks. The minimum total duration of the NMES in the included studies are in line with the studies which show significant improvements in peripheral muscle function [13],[14].

### Changes in enzyme activity following NMES

Activity of oxidative enzymes generally increased significantly following 6 weeks of LF-NMES. The increase in oxidative enzyme activity was accompanied with an improved resistance to fatigue [52], [53] and an improvement in functional exercise capacity [47]. Compared with a minimum of 6 weeks of endurance cycling training [60], [61], the absolute and relative improvement in CS activity after NMES is lower.

Levels of glycolytic enzymes did not change or decreased following LF-NMES. These results are comparable with endurance training in healthy young men [62] and in patients with COPD [63]. In healthy volunteers levels of oxidative enzymes increased following HF-NMES [40], [48]. These adaptations are more endurance-specific. Collins and colleagues recently showed that the use of a wide pulse (1 ms), high frequency (80–100 Hertz) and a low intensity might favour the recruitment of fatigue-resistant motor units (according to the Henneman's size principle) [64]. This combination of stimulation parameters could also be relevant for increasing oxidative capacity. However, they used a wide pulse and low stimulation intensity whereas the included trials in the present review [40], [48] used narrow pulses and intensities at the maximum toleration level. As the consequence, the corresponding increase of oxidative enzymes is likely due to the non-selective recruitment of both type IIx and type I fibers during HF-NMES [17]. Another study limitation is that only one study (in healthy volunteers) with a very small sample size [40] studied glycolytic enzymes following HF-NMES. In the sedentary group the downregulation of glycolytic enzymes is highly consistent with the fast-to-slow MHC isoform shift as slow fibers mostly have an oxidative metabolism and type IIx fibers mostly have a glycolytic metabolism. Additionally, glycolytic enzyme content is known to increase in the order of slow oxidative, fast oxidative, glycolytic, fast glycolytic fibers [65]. However, the small sample size is too limited (n = 10) to provide an answer on the hypothesis that HF-NMES increases glycolytic capacity.

### Changes in skeletal muscle fiber type composition following NMES

Changes in type I and IIa fiber proportion were variable following HF-NMES. Resistance training resulted in no changes in type I fibers and an increase of type II fibers [7], [66]. However, based on the results of the present systematic review changes in type IIa and type IIx fibers following regular resistance training programs [67] cannot be compared with HF-NMES. Following LF-NMES type I and IIa fibers increased. Endurance training programs also resulted in an increase of type I and type IIa fibers and a decrease of type IIx fibers [4], [5], [68]. However, considering the small number of studies and heterogeneity in NMES protocols and study populations it is difficult to draw relations between LF-NMES and endurance training.

### Changes in muscle size following NMES

In 50% of the HF-NMES studies muscle fiber size increased significantly. The increased CSA was accompanied with an increased muscle strength [40]–[42], [49]. Changes in total muscle fiber size following LF-NMES are conflicting. Differences in the LF-NMES studies which could possible explain the conflicting results are the study population (age, healthy volunteers versus patients with chronic organ failure), measurement of muscle fiber size or intensity of NMES.

These results are in line with previous studies concluding that muscle fiber size increased less in subjects who performed endurance training than in strength training [69], [70]. Conflicting results for the changes in muscle fiber size in HF-NMES could be related to the intensity of the training. In two trials [38], [51] reporting no changes in muscle fiber size, the stimulus intensity varied from “a comfortable stimulus” [51] to 25 mA [38] compared with a stimulus at the maximum tolerable level in the trials with an increased muscle fiber size [40]–[42], [48]. Moreover, Vivodtzev and colleagues showed that gains in muscle strength were proportional to the increase in pulse amplitude during the training program and to the final pulse amplitude of training [54]. The impact of NMES is also dependent on the training duration. It is well known that long training duration is needed to induce muscle hypertrophy [71], however the total duration time in the HF-NMES trials ranged from 2 [45] to 54 hours [50].

### Recommendations

Overall, LF-NMES seems to improve oxidative phenotype (oxidative enzyme capacities, type I/IIa fibers). However, some of the results of the included trials are difficult to compare and cannot be generalized. Besides the heterogeneity in NMES protocols and study designs, the number of study populations is limited and varies among the trials, from well-trained healthy volunteers [40] to patients with severe chronic organ failure [38], [54] or severe orthopedic problems [50], [55]. Stimulation variables (i.e. pulse amplitude, session time and number of sessions) might have influenced the number of muscle fibers recruited during NMES, the motor unit recruitment order and the types of the recruited muscle fibers [18], [72].

Therefore, future trials are needed to determine the optimal settings of NMES, such as stimulation frequency (HF-NMES or LF-NMES), session time, pulse amplitude and electrodes (number, size and location) in healthy (i.e. athletes) and in diseased people (i.e. COPD, CHF, orthopedic problems). These trials should not only study the effects of NMES *versus* volitional training, but also study the superimposed effects of NMES on volitional training.

Based on the results of the present systematic review, randomized controlled trials using concealed allocation, blinded therapists, blinded participants and blinded outcome assessors are recommended. Additionally, studies should focus on larger study populations, including both genders and a broad range in age. These studies should not include only healthy people but also people who are unable to perform or complete volitional exercise training programs. Finally, safety should be added to new randomized controlled trials as secondary outcome.

## Conclusion

NMES seems to be a training modality resulting in changes in oxidative enzyme activity, skeletal muscle fiber type and skeletal muscle fiber size. A more formal meta-analysis would be a more rigorous way to analyze the current data, but is not possible at this time. Indeed, the small sample sizes, the variance in study populations, the non-randomized controlled study designs, the variance in primary outcomes and the large heterogeneity in NMES protocols are major methodological limitations which may limit the external validity of the current findings. Therefore, it is difficult to draw definitive conclusions about the effects of stimulation frequencies on muscular changes. This systematic review, however, will help generate discussion in the field that would lead to a consensus in study design that would permit a meta-analysis in the future.

A better understanding of metabolic and structural changes following NMES is of particular clinical interest as it will increase its applicability in specific populations who are not able to perform regular exercise training. Therefore, future well-designed, randomized controlled trials with larger study samples are needed to determine the optimal NMES settings (i.e. electrode placement, stimulation frequency and pulse amplitude) to achieve endurance or resistance training-like adaptations. The actual stimulation parameters, session time, total time and changes in NMES pulse intensity over time should be reported to enable comparisons between studies and to facilitate the further development and implementation of NMES.

## Supporting Information

Appendix S1 **Searchstring Medline/PubMed 02-03-2012.**
(DOC)

Checklist S1 **PRISMA Checklist.**
(DOC)
