# Supplementary material for: Metabolic and Structural Changes in Lower-Limb Skeletal Muscle Following Neuromuscular Electrical Stimulation: A Systematic Review
Source: PLoS One. 2013 Sep 3;8(9):e69391. doi: 10.1371/journal.pone.0069391 (PMC3760845; doi:10.1371/journal.pone.0069391)
Supplement: Appendix S1 — Searchstring Medline/PubMed 02-03-2012. (DOC) [file pone.0069391.s001.doc]

**Appendix SI**

Searchstring Medline/PubMed 02-03-2012:

(“neuromuscular electrical stimulation” OR “functional electrical stimulation” OR “muscle stimulation” OR “transcutaneous electrical stimulation” OR electrophysiology OR electrotherapy OR neurostimulation OR "electric stimulation" OR "electrical stimulation" OR elektromyostimulation OR electromyostimulation OR electrostimulation OR "muscle stimulation" OR NMES OR FES OR TENS OR EMS) AND (quadriceps OR vastus OR "knee extensor" OR "knee flexor" OR "triceps surae" OR gastrocnemius OR calf OR tibialis OR soleus OR hamstring) AND (muscle AND ("Muscle Fibers, Fast-Twitch" OR "Muscle Fibers, Slow-Twitch" OR biopsy OR biopsies OR "Enzymes" OR enzymes OR fibers OR fibres OR "muscle mass" OR volume OR "cross sectional area" OR CSA)) AND (“randomized controlled trial” OR “controlled clinical trial” OR OR “clinical trial” OR trial OR RCT OR CCT OR weeks OR wk OR week OR month OR training OR rehabilitation) NOT (review [pt] OR letter [pt] OR animal OR rat OR mouse OR mice OR equine OR horse)
